# Supplementary material for: Ethnic Minorities’ Experiences of Cardiac Rehabilitation: A Scoping Review
Source: Healthcare (Basel). 2023 Mar 4;11(5):757. doi: 10.3390/healthcare11050757 (PMC10000677; doi:10.3390/healthcare11050757)
Supplement: Supplementary file 1 [file healthcare-11-00757-s001.zip › Inclusion criteria.pdf]

Inclusion Exclusion Criteria

**Inclusion criteria**	**Exclusion criteria**
All Articles in English	Articles not in English language
Qualitative Studies	Quantitative studies
Ethnic minority individuals including individuals of American Indian, Asian, Black or African American, Hispanic and White having origins in any of the original peoples of Europe, the middle East or North Africa	Studies not explicitly outlining the experiences of ethnic minority individuals.
Studies with explicit mention of CR reporting on an aspect of Cardiovascular rehabilitation	Studies not explicitly reporting on an aspect of cardiovascular rehabilitation.
Studies reporting primary data	Studies not reporting primary data (e.g. systematic reviews.
