# Supplementary material for: Ethnic Minorities’ Experiences of Cardiac Rehabilitation: A Scoping Review
Source: Healthcare (Basel). 2023 Mar 4;11(5):757. doi: 10.3390/healthcare11050757 (PMC10000677; doi:10.3390/healthcare11050757)
Supplement: Supplementary file 1 [file healthcare-11-00757-s001.zip › List of Excluded Studies with reasons for exclusion.pdf]

List of Excluded Studies with reasons for exclusion *(n-33)*

Limited focus on study outcome	*(n-7)*
Gender Specific outcome	*(n=1)*
Type of Study	*(n=6)*
Limited focus on the study population outcome	*(n=18)*
Same study participants used in study	*(n=1)*

References	Reason for Exclusion
1. Midence, L., Mola, A., Terzic, C. M., Thomas, R. J., & Grace, S. L. (2014). Ethnocultural diversity in cardiac rehabilitation. *Journal of Cardiopulmonary Rehabilitation and Prevention*, *34*(6), 437–444. https://doi.org/10.1097/HCR.0000000000000089	Type of study
2. Valencia, H. E., Savage, P. D., & Ades, P. A. (2011). Cardiac Rehabilitation Participation in Underserved Populations. https://doi.org/10.1097/HCR.0b013e318220a7da	Type of study
3. Vanzella, L. M., Oh, P., Pakosh, M., & Ghisi, G. L. M. (2021). Barriers to Cardiac Rehabilitation in Ethnic Minority Groups: A Scoping Review. *Journal of Immigrant and Minority Health*, *23*(4), 824–839. https://doi.org/10.1007/s10903-021-01147-1	Type of study
4. Zhang, L., Gallagher, R., Ding, D., & Neubeck, L. (2018). Self-management Following a Cardiac Event in People of Chinese Ethnicity Living in Western Countries: A Scoping Review. *Journal of Immigrant and Minority Health*, *20*(3), 744–754. https://doi.org/10.1007/s10903-017-0584-6	Type of study
5. Gupta, S. S., Aroni, R., & Teede, H. (2017). Experiences and Perceptions of Physical Activity among South Asian and Anglo-Australians with Type 2 Diabetes or Cardiovascular Disease: Implications for Clinical Practice. *Qualitative Health Research*, *27*(3), 391–405. https://doi.org/10.1177/1049732316660690	Limited focus on study outcome
6. Lord, H., MacPhail, C., Cherry, J., & Fernandez, R. (2021). Perceptions of Aboriginal and Torres Strait Islander Australians toward cardiovascular primary prevention programs: A qualitative systematic review. Public Health Nursing, 38(2), 197–211. https://doi.org/10.1111/phn.12837	Limited focus on study outcome

7. Ellis, K. R., Young, T. L., Carthron, D., Simms, M., McFarlin, S., Davis, K. L., Dave, G., Corbie-Smith, G., & Cené, C. (2019). Perceptions of Rural African American Adults About the Role of Family in Understanding and Addressing Risk Factors for Cardiovascular Disease. American Journal of Health Promotion, 33(5), 708–717. https://doi.org/10.1177/0890117118799574	Limited focus on the study population outcome
8. Bäck, M., Öberg, B., & Krevers, B. (2017). Important aspects in relation to patients' attendance at exercise-based cardiac rehabilitation - facilitators, barriers, and physiotherapist's role: A qualitative study. BMC Cardiovascular Disorders, 17(1), 1–10. https://doi.org/10.1186/s12872-017-0512-7	Limited focus on the study population outcome
9. Evans, A. B., & Crust, L. (2015). 'Some of these people aren't as fit as us …': experiencing the ageing, physically active body in cardiac rehabilitation. Qualitative Research in Sport, Exercise and Health, 7(1), 13–36. https://doi.org/10.1080/2159676X.2014.908945	Limited focus on the study population outcome
10. Hanna, A., Yael, E. M., Hadassa, L., Iris, E., Eugenia, N., Lior, G., Carmit, S., & Liora, O. (2020). ``It's up to me with a little support'' – Adherence after myocardial infarction: A qualitative study. International Journal of Nursing Studies, 101, 103416. https://doi.org/10.1016/j.ijnurstu.2019.103416	Limited focus on the study population outcome
11. Jackson, A. M., McKinstry, B., Gregory, S., & Amos, A. (2012). A qualitative study exploring why people do not participate in cardiac rehabilitation and coronary heart disease self-help groups, and their rehabilitation experience without these resources. Primary Health Care Research & Development, 13(1), 30–41. https://doi.org/10.1017/S1463423611000284	Limited focus on the study population outcome
12. Lesage-Moussavou-Nzamba, M., Houle, J., & Trudeau, F. (2020). Participants Perspectives of a Primary Exercise-Based Prevention Program for Cardiac Patients: A Prepost Intervention Qualitative Case Study. Rehabilitation Research and Practice, 2020. https://doi.org/10.1155/2020/6215428	Limited focus on the study population outcome
13. Madden, M., Furze, G., & Lewin, R. J. P. (2011). Complexities of patient choice in cardiac rehabilitation: Qualitative findings. Journal of Advanced Nursing, 67(3), 540–549. https://doi.org/10.1111/j.1365-2648.2010.05509.x	Limited focus on the study population outcome
14. Rouleau, C. R., King-shier, K. M., Tomfohr-madsen, L. M., Aggarwal, S. G., Arena, R., Campbell, T. S., Rouleau, C. R., King-shier, K. M.,	Limited focus on the study population outcome

Tomfohr-madsen, L. M., Aggarwal, S. G., Arena, R., & Campbell, T. S. (2017). A qualitative study exploring factors that influence enrollment in outpatient cardiac rehabilitation. Disability and Rehabilitation, 0(0), 000. https://doi.org/10.1080/09638288.2016.1261417	
15. Saeidi, M., Soroush, A., Komasi, S., Moemeni, K., & Heydarpour, B. (2015). Attitudes toward cardiovascular disease risk factors among patients referred to a cardiac rehabilitation center: Importance of psychological attitudes. Shiraz E Medical Journal, 16(7), 1–3. https://doi.org/10.17795/semj22281	Limited focus on the study population outcome
16. Schröder, S. L., Fink, A., & Richter, M. (2018). Socioeconomic differences in experiences with treatment of coronary heart disease: A qualitative study from the perspective of elderly patients. BMJ Open, 8(11), 1–8. https://doi.org/10.1136/bmjopen-2018-024151	Limited focus on the study population outcome
17. Hooman Shahsavari1, Mohsen Shahriari2, Nasrollah Alimohammadi<br>Motivational factors of adherence to cardiac rehabilitation Hooman Shahsavari1, Mohsen Shahriari2, Nasrollah Alimohammadi	Limited focus on the study population outcome
18. Ades, P. A., Khadanga, S., Savage, P. D., & Gaalema, D. E. (2022). Enhancing participation in cardiac rehabilitation: Focus on underserved populations. *Progress in Cardiovascular Diseases*, *70*, 102–110. https://doi.org/10.1016/j.pcad.2022.01.003	Type of study
19. Chauhan, U. et al. (2010) 'Improving Care in Cardiac Rehabilitation for Minority Ethnic Populations', European Journal of Cardiovascular Nursing, 9(4), pp. 272–277. doi: 10.1016/j.ejcnurse.2010.03.004.	Limited focus on the study population outcome
20. Brown, A. (2010). Acute Coronary Syndromes in Indigenous Australians: Opportunities for Improving Outcomes Across the Continuum of Care. Heart Lung and Circulation, 19(5–6), 325–336. https://doi.org/10.1016/j.hlc.2010.02.011	Limited focus on the study population outcome
21. Galdas, P. M., Oliffe, J. L., Wong, S. T., Ratner, P. A., Johnson, J. L., & Kelly, M. T. (2012). Canadian Punjabi Sikh men's experiences of lifestyle changes following myocardial infarction: Cultural connections. Ethnicity and Health, 17(3), 253–266. https://doi.org/10.1080/13557858.2011.610440	Gender specific

22. Grewal, K., Leung, Y. W., Safai, P., Stewart, D. E., Anand, S., Gupta, M., Parsons, C., & Grace, S. L. (2010). Access to cardiac rehabilitation among South-asian patients by referral method: A qualitative study. Rehabilitation Nursing, 35(3), 106–112. https://doi.org/10.1002/j.2048-7940.2010.tb00285.x	Limited focus on the study population outcome
23. Gupta, S. S., Aroni, R., & Teede, H. (2017). Experiences and Perceptions of Physical Activity among South Asian and Anglo-Australians with Type 2 Diabetes or Cardiovascular Disease: Implications for Clinical Practice. *Qualitative Health Research*, *27*(3), 391–405. https://doi.org/10.1177/1049732316660690	Limited focus on study outcome
24. Jin, K., Neubeck, L., Koo, F., Ding, D., & Gullick, J. (2020). Understanding Prevention and Management of Coronary Heart Disease Among Chinese Immigrants and Their Family Carers: A Socioecological Approach. Journal of Transcultural Nursing, 31(3), 257–266. https://doi.org/10.1177/1043659619859059	Limited focus on study outcome
25. Maddocks, S., & Cobbing, S. (2017). Patients' experiences of and perspectives on phase 1 cardiac rehabilitation after coronary artery bypass graft surgery. *Physiotherapy Canada*, *69*(4), 333–340. https://doi.org/10.3138/ptc.2016-39GH	Limited focus on study outcome
26. Nielsen, L. S., Angus, J. E., Lapum, J., Dale, C., Kramer-Kile, M., Abramson, B., Marzolini, S., Oh, P., Price, J., & Clark, A. (2012). "I can't just follow any particular textbook": Immigrants in cardiac rehabilitation. Journal of Advanced Nursing, 68(12), 2719–2729. https://doi.org/10.1111/j.1365-2648.2012.05975.x	Limited focus on the study population outcome
27. Piepoli, M. F., Corrà, U., Benzer, W., Bjarnason-Wehrens, B., Dendale, P., Gaita, D., McGee, H., Mendes, M., Niebauer, J., Zwisler, A. D. O., & Schmid, J. P. (2010). Secondary prevention through cardiac rehabilitation: From knowledge to implementation. A position paper from the cardiac rehabilitation section of the European association of cardiovascular prevention and rehabilitation. *European Journal of Cardiovascular Prevention and Rehabilitation*, *17*(1), 1–17. https://doi.org/10.1097/HJR.0b013e3283313592	Type of paper
28. Poh, R., Ng, H. N., Loo, G., Ooi, L. S., Yeo, T. J., Wong, R., & Lee, C. H. (2015). Cardiac Rehabilitation after Percutaneous Coronary Intervention in a Multiethnic Asian Country:	Limited focus on the study population outcome

Enrollment and Barriers. Archives of Physical Medicine and Rehabilitation, 96(9), 1733–1738. https://doi.org/10.1016/j.apmr.2015.05.020	
29. Scotto, C. J., Waechter, D., & Rosneck, J. (2011). Factors affecting program completion in phase II cardiac rehabilitation. *Canadian Journal of Cardiovascular Nursing = Journal Canadien En Soins Infirmiers Cardio-Vasculaires*, *21*(2), 15–20.	Limited focus on study outcome
30. Tuomisto, S., Koivula, M., Åstedt-Kurki, P., & Helminen, M. (2018). Family involvement in rehabilitation: Coronary artery disease—patients' perspectives. Journal of Clinical Nursing, 27(15–16), 3020–3031. https://doi.org/10.1111/jocn.14494	Limited focus on the study population outcome
31. Vilchinsky, N., Reges, O., Leibowitz, M., Khaskia, A., Mosseri, M., & Kark, J. D. (2018). Symptoms of Depression and Anxiety as Barriers to Participation in Cardiac Rehabilitation Programs among Arab and Jewish Patients in Israel. Journal of Cardiopulmonary Rehabilitation and Prevention, 38(3), 163–169.	Limited focus on study outcome
32. Darr, A., Astin, F., & Atkin, K. (2008). Causal attributions, lifestyle change, and coronary heart disease: Illness beliefs of patients of South Asian and European origin living in the United Kingdom. Heart and Lung: Journal of Acute and Critical Care, 37(2), 91–104. https://doi.org/10.1016/j.hrtlng.2007.03.004	Same study participants used in study
33. Yates, B. C., Vazquez Hernandez, M. L., Rowland, S. A., Bainter, D. E., Schulz, P., & Hanson, C. K. (2018). A Qualitative Study of Experiences of Participants in Cardiac Rehabilitation. *Journal of Cardiopulmonary Rehabilitation and Prevention*, *38*(4), E6–E9. https://doi.org/10.1097/HCR.0000000000000317	Limited focus on the study population outcome
