# Supplementary material for: Ethnic Minorities’ Experiences of Cardiac Rehabilitation: A Scoping Review
Source: Healthcare (Basel). 2023 Mar 4;11(5):757. doi: 10.3390/healthcare11050757 (PMC10000677; doi:10.3390/healthcare11050757)
Supplement: Supplementary file 1 [file healthcare-11-00757-s001.zip › Screening and Selection Tool.pdf]

<u>Screening and Selection Tool</u>

Review Question: How effective is Cardiac Rehabilitation programs for the ethnic minority patients based on individuals' experiences.

Population - Male and Female ethnic minority patients aged 18 > who experience any clinical cardiac injury/event

Intervention -Any Cardiac rehabilitation programs / Activities/ intervention /services given for the after care and recovery of any cardiac injury/ event which requires cardiac rehabilitation

Comparison-N/A

Outcome- Personal experiences of attending CR services

Setting- Any clinical, community, hospital, health program, care centre, institutions environments

Study Designs- Qualitative study designs only

Reviewer Name and Date: Author 1, 20/10/22

Title of study and citation: 1. Dilla, D., Ian, J., Martin, J., Michelle, H., & Felicity, A. (2020). "I don't do it for myself, I do it for them": A grounded theory study of South Asians' experiences of making lifestyle change after myocardial infarction. Journal of Clinical Nursing, 29(19–20), 3687–3700. https://doi.org/10.1111/jocn.15395

Appendix B

Screening and Selection Tool Part B

*Tick the appropriate box when screening and selecting studies for inclusion (Yes, No Unsure or Exclude)

**Population**	Include if the study involves Male and Female ethnic minority patients aged 18 > who experience any clinical cardiac injury/event<br>**Include □√**	**Unsure**<br>□	Exclude if the study involves Male and Female patients originating from non-ethnic backgrounds aged below 18 years who experience other non-cardiac injury/event.<br>**Exclude □**
**Intervention**	Include if the study involves attendees to Cardiac rehabilitation programs / Activities/ intervention /services given for the after care and recovery of any cardiac event which requires cardiac rehabilitation<br>**Include □√**	**Unsure**<br>□	Exclude if the study does not involve attendees to any Cardiac rehabilitation programs / Activities/ intervention or services given for the after care and recovery of any cardiac event which requires cardiac rehabilitation<br>**Exclude □**
**Comparison**	**NA**	**NA**	**NA**
**Outcomes**	Include if the study discusses personal experiences of attending CR services for ethnic minority patients<br>**Include □√**	**Unsure**<br>□	Exclude if the study does not discuss or provides limited or no detail of personal experiences of attending CR services for ethnic minority patients or if the outcome of the study is age and gender specific.<br>**Exclude □**
**Setting**	Include if the setting of study has been conducted in any country in any clinical, community, hospital, health program, care centre or institutions environments<br>**Include □√**	**Unsure**<br>□	Exclude of the setting of study has not been conducted in any clinical, community, hospital, health program, care centre or institutions environments or does not state<br>**Exclude □**
**Study Design**	Include if the study design provides qualitative data<br>**Include □√**	**Unsure**<br>□	Exclude if the study design does not provide qualitative data.<br>**Exclude □**
**Overall Decision**	**Include □√**		**Exclude □**
**Notes**	Limited data on personal CR experiences		

Template example revised and taken from Boland, A., Cherry, M.G. and Dickson, R. (2017) *Doing A Systematic Review A Students Guide.* 2nd edn. London: Sage Publications.
